# *flp*-32 Ligand/Receptor Silencing Phenocopy Faster Plant Pathogenic Nematodes

**DOI:** 10.1371/journal.ppat.1003169

**Published:** 2013-02-28

**Authors:** Louise E. Atkinson, Michael Stevenson, Ciaran J. McCoy, Nikki J. Marks, Colin Fleming, Mostafa Zamanian, Tim A. Day, Michael J. Kimber, Aaron G. Maule, Angela Mousley

**Affiliations:** 1 Molecular Biosciences-Parasitology, Institute for Global Food Security, School of Biological Sciences, Queen's University Belfast, Belfast, United Kingdom; 2 Agri-Food and Biosciences Institute, Belfast, United Kingdom; 3 Institute of Parasitology, McGill University, Ste. Anne de Bellevue, Quebec, Canada; 4 Department of Biomedical Sciences, Iowa State University, Ames, Iowa, United States of America; North Carolina State University, United States of America

## Abstract

Restrictions on nematicide usage underscore the need for novel control strategies for plant pathogenic nematodes such as *Globodera pallida* (potato cyst nematode) that impose a significant economic burden on plant cultivation activities. The nematode neuropeptide signalling system is an attractive resource for novel control targets as it plays a critical role in sensory and motor functions. The FMRFamide-like peptides (FLPs) form the largest and most diverse family of neuropeptides in invertebrates, and are structurally conserved across nematode species, highlighting the utility of the FLPergic system as a broad-spectrum control target. *flp*-32 is expressed widely across nematode species. This study investigates the role of *flp*-32 in *G. pallida* and shows that: (i) *Gp-flp*-32 encodes the peptide AMRNALVRFamide; (ii) *Gp-flp*-32 is expressed in the brain and ventral nerve cord of *G. pallida*; (iii) migration rate increases in *Gp-flp*-32-silenced worms; (iv) the ability of *G. pallida* to infect potato plant root systems is enhanced in *Gp-flp*-32-silenced worms; (v) a novel putative *Gp-flp*-32 receptor (*Gp-flp*-32R) is expressed in *G. pallida*; and, (vi) *Gp-flp*-32R-silenced worms also display an increase in migration rate. This work demonstrates that *Gp-flp*-32 plays an intrinsic role in the modulation of locomotory behaviour in *G. pallida* and putatively interacts with at least one novel G-protein coupled receptor (*Gp-flp*-32R). This is the first functional characterisation of a parasitic nematode FLP-GPCR.

## Author Summary

Plant pathogenic nematodes compromise plant health and productivity globally and are an increasing problem due to the lack of efficient control measures. The nematode nervous system depends heavily on small proteins (neuropeptides) for communication between nerve cells and other nerve cells or other cell types. The disruption of neuropeptide signalling would dysregulate normal behaviour, offering an attractive approach to parasite control. One major group of nematode neuropeptides are the FMRFamide-like peptides (FLPs) that alter nematode behaviour by acting on receptors designated G-protein coupled receptors (GPCRs). GPCRs are attractive targets based on their potential ‘druggability;″ indeed they are targets for many human medicines. This study investigates the functional biology of *flp*-32, a commonly expressed nematode *flp*, and a novel FLP-32 receptor in a plant pathogenic nematode of major agricultural importance, *Globodera pallida*. We show that FLP-32 occurs widely in these parasites and interacts with a novel FLP-32 receptor to modulate their behaviour, affecting their movement and the rate at which they infect host plants. These data indicate that chemicals that activate the FLP-32 receptor in these parasites could effectively slow the worms, potentially making them less successful parasites. The conservation of the FLP-32 ligand and receptor across many different nematode parasites adds to its appeal as a potential target for broad-spectrum parasite control.

## Introduction

Plant pathogenic nematodes (PPNs) impose a significant economic burden on global crop cultivation resulting in estimated losses of at least $118 billion per year [1]. The control of PPNs relies heavily on nematicides, basic crop rotation approaches, and the use of resistant crop cultivars; significantly many nematicides have diminishing utility as a consequence of their environmental toxicity. Consequently, global crop production remains under threat from PPNs for which no effective management strategies currently exist. While the PPN problem results from an absence of effective, legal control methods, deficiencies in animal and human parasite therapies are associated with escalating reports of resistance to chemotherapeutics [2]. Thus, while focused control of PPNs alone has significant merit, the identification of broad spectrum drug targets and chemotherapies which combat diverse nematode infections is highly desirable.

Many of the most useful anti-nematode chemotherapeutics target neuromuscular signalling, compromising nerve-muscle function to impair normal parasite biology. One approach to novel drug target discovery in nematodes is the interrogation of alternative, unexploited , facets of this already proven repository (see [3] for review). Within the neuropeptidergic system, motor functions (reproduction, feeding and locomotion) are known to be modulated by FMRFamide-like peptides (FLPs); the nematode FLPergic system remains unexploited for parasite control (see [3]–[9] for review). The potential of FLPergic signalling as a control target resource embedded within nematode neuromuscular functionality has been highlighted [3].

FLP structural diversity hinges upon a conserved C-terminal tetrapeptide motif X-X_o_ –Arg-Phe-NH_2_ (X represents any amino acid and X_o_ any hydrophobic amino acid except cysteine; [10]). BLAST-based bioinformatics has revealed a high degree of inter-species FLP conservation across nematode clades, with a number of species possessing a FLP complement with comparable complexity to the model nematode *C. elegans*, which expresses 31 *flps* encoding >70 distinct peptides [10]–[12]. If FLP structural conservation is mirrored by conserved function then the potential broad-spectrum utility of drugs directed against FLP signalling targets would be enhanced.

While FLPs themselves are of limited control value, they do facilitate the identification of appealing targets, such as the G-protein coupled receptors (GPCRs) by which the majority mediate their biological effects. Although 12 FLP GPCRs have been deorphanised in *C. elegan*s [13], [14], none have been characterised in nematode parasites; whilst *flp-1*, *-8*, and *-18* peptides have been shown to interact with a latrophillin-like GPCR in *Haemonchus contortus*, their affinity for this receptor was low [15].

The *C. elegans* VRFamide receptor 1 (C26F1.6) is potently activated by two peptides, TPMQRSSMVRFamide and AMRNALVRFamide [13]. In *C. elegans* a single copy of AMRNALVRFamide is encoded by *flp*-11 and *flp*-32, however in *G. pallida* this peptide is encoded on *flp*-32 only [10]. Subsequent BLAST interrogation of available nematode genome, transcriptome and expressed sequence tag (EST) datasets has revealed that *flp*-32 is conserved in at least 16 nematode species, across two nematode clades, encompassing a number of contrasting lifestyles ([11]; unpublished data). Such conservation suggests that FLP-32 may modulate functionally important signalling pathways within the neuromuscular signalling system; building a pan-phylum picture of *flp*-32 biology in multiple pathogenic nematode species will generate valuable information on the biological role of this peptide and validate its potential as a broad spectrum control target.

Here we report the functional characterisation of *Gp-flp*-32 and a putative *Gp-flp*-32 receptor (*Gp-flp*-32R) from the potato cyst nematode (PCN) *G. pallida*, a pathogenic nematode which is readily amenable to reverse genetic techniques [7], and boasts a completed genome sequence (http://www.sanger.ac.uk/cgi-bin/blast/submitblast/g_pallida). We also describe a novel *in vivo* reverse genetics approach to putative FLP receptor deorphanisation in parasitic nematodes.

## Results/Discussion

### *Gp-flp*-32 encodes a single peptide – AMRNALVRFamide

**Figure 1:**
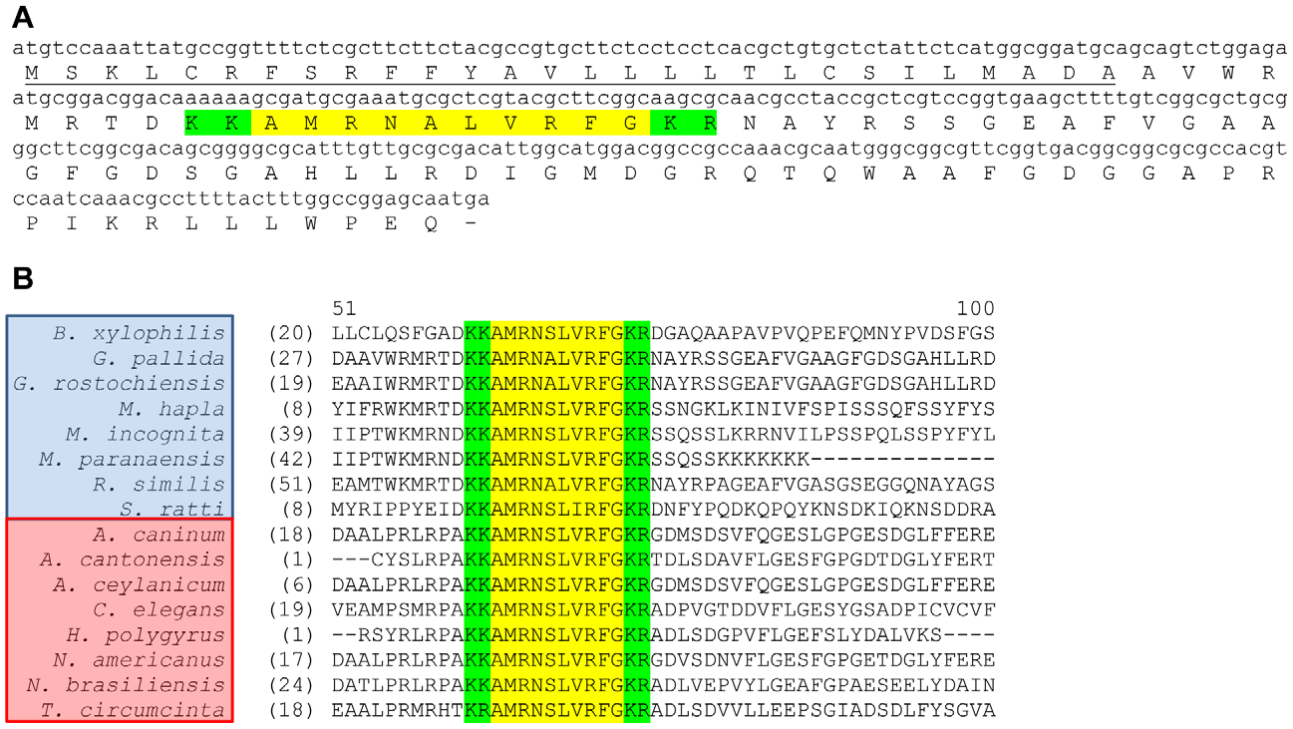
*Globodera pallida flp*-32 (*Gp-flp*-32) encodes a single peptide (AMRNALVRFG) and is expressed in at least 16 nematode species. *Gp-flp*-32 encodes one FMRFamide-like peptide, AMRNALVRFG (highlighted in yellow; A), flanked by dibasic cleavage sites (KK/KR; highlighted in green; A), and preceded by a predicted 28 amino acid N-terminal signal peptide (underlined; A). At least 16 nematode species from clades IV (species boxed in blue) and V (species boxed in red) express *flp-32*, encoding the single highly conserved AMRNA/SLVRFG FLP-32 peptide (highlighted in yellow; B), flanked by conserved dibasic cleavage sites (KK/KR; highlighted in green; B). *B. xylophilis: Bursaphelenchus xylophilus; G. pallida: Globodera pallida; G. rostochiensis: Globodera rostochiensis; M. hapla*: *Meloidogyne hapla*; *M. incognita*: *Meloidogyne incognita*; *M. paranaensis: Meloidogyne paranaensis; R. similis: Radopholus similis; S. ratti: Strongyloides ratti; A. caninum: Ancylostoma caninum; A. cantonensis: Ancylostoma cantonensis; A. ceylanicum; Ancylostoma ceylanicum; C. elegans: Caenorhabditis elegans; H. polygyrus: Heligmosomoides polygyrus; N. americanus*: *Necator americanus; N. brasiliensis: Nippostrongylus brasiliensis; T. circumcincta: Teladorsagia circumcincta*.

*flp*-32 is expressed in at least 16 nematode species where it encodes a highly conserved peptide with a characteristic VRFamide C-terminal motif, AMRN(A/S)LVRFG (see Fig. 1B). Previous interrogation of *G. pallida* ESTs [11] identified a transcript encoding a putative FLP-32-like peptide (GenBank accession number CV578361), which was used in this study to aid PCR confirmation of the full length *Gp-flp*-32 transcript. Primers designed to confirm the open reading frame of *Gp-flp*-32 generated a 321 nucleotide cDNA sequence (GenBank accession number JQ685131), encoding a 107 amino acid (aa) protein (Fig. 1A). The confirmed *Gp-flp*-32 aa sequence encodes a single copy of the FLP-32 peptide, AMRNALVRFG, flanked at both ends by dibasic residues (KK/KR), and a 28 aa signal peptide (see Fig. 1A; [16]). Further interrogation of the *G. pallida* EST database (GenBank) and genome assembly (Wellcome Trust Sanger Institute, *G. pallida* November 2010 supercontig assembly) in April-August 2011 did not reveal additional AMRNALVRFG encoding transcripts.

### *Gp-flp*-32 is widely expressed in the nervous system of *G. pallida*

**Figure 2:**
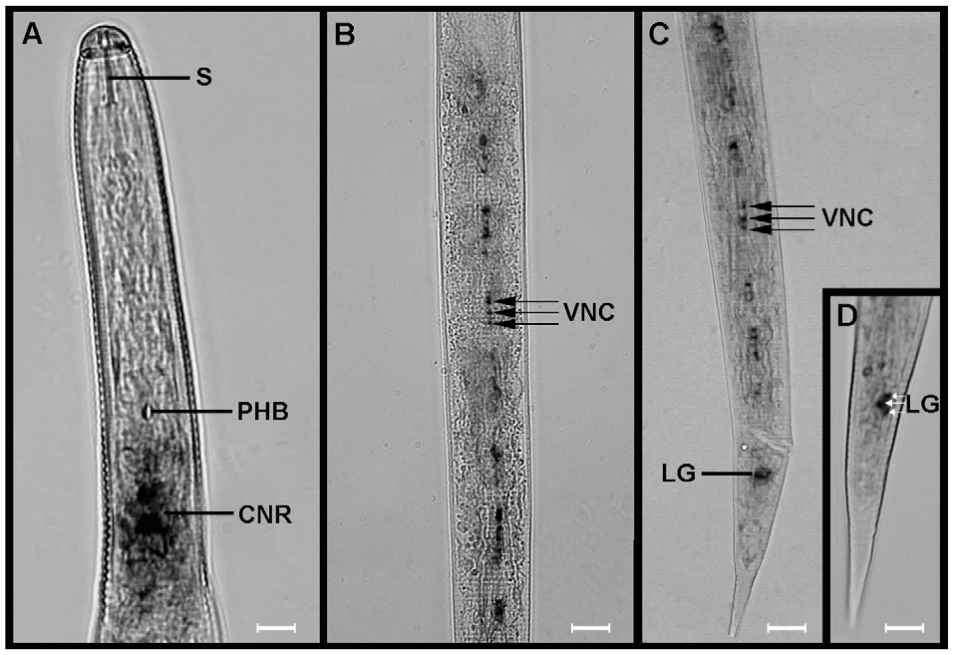
*Globodera pallida flp*-32 (*Gp-flp*-32) is expressed in the brain and ventral nerve cords of pre-parasitic juveniles (J2s). Light microscopy *in situ* hybridisation images show *Gp-flp*-32 gene expression in the circumpharyngeal nerve ring (CNR) posterior to the stylet (S) and the pharyngeal bulb (PHB) of pre-parasitic juvenile (J2) *G. pallida* (A). *Gp-flp*-32 is also expressed in repeating groups of three to four cell bodies located in the ventral nerve cord (VNC; B and C) which runs posteriorly to the tail where a cluster of three cell bodies also expressing *Gp-flp*-32 are located in the region of the lumbar ganglia (LG; C and D). Scale bars: A–C = 12 µm; D = 5 µm.

*Gp-flp*-32 expression, visualised by the hybridisation of a 201 base pair (bp) probe, was identified both within and connecting the anterior and posterior regions of the nematode (see Fig. 2A–D). Staining was evident in the circumpharyngeal nerve ring (CNR), and within multiple distinct cell bodies in the ventral nerve cord (VNC) and lumbar ganglia (LG) (see Fig. 2A–D). Staining within the CNR was diffuse with no specific neuronal cell bodies staining strongly (Fig. 2A); this pattern was evident in the majority of specimens (>90%) treated with the antisense probe, and is similar to a diffuse ISH staining pattern previously reported in the *G. pallida* CNR for *flp-6* (KSAYMRFG; [17]). In contrast, staining in the VNC was characterised by groups of three to four distinct and strongly reactive cell bodies spaced at regular intervals along the nerve cord, beginning posterior to the CNR and running into the tail (Fig. 2B). Although slightly variable, most specimens exhibited approximately six to eight groups of cells in the VNC, with as many as 18 cell bodies visible at any one time (see Fig. 2B and C). While unequivocal assignment of neuronal cell bodies is difficult, in this scenario it is likely that they belong to VD and/or DD motor neurons which possess cell bodies in the VNC of *C. elegans*, where they have been shown to express a VRFamide-like *flp* gene encoding peptides similar to that encoded by *Gp-flp-*32 [18]. VD neurons are a set of 13 motor neurons which innervate ventral muscle and have cell bodies in the VNC, while DD are a set of six motor neurons (pre-synaptic to VD), which also possess cell bodies in the VNC, but instead innervate dorsal muscle [19], [20]. Together this amounts to 19 identifiable cell bodies within the VNC, and while this number is known to vary in *C. elegans* according to developmental stage [19], it is similar to the 18 cell bodies visible within the VNC of *G. pallida* following *Gp-flp*-32 antisense probe hybridisation.

Defined staining was also identified in a tightly associated group of three cell bodies close to the tip of the tail in the region of the LG, a group of cell bodies which cluster together posterior to the pre-anal ganglia (PAG) and the termination of the VNC (see Fig. 2D). Again whilst unequivocal identification of cells is difficult in such a tightly packed ganglion, *C. elegans* neurons which possess cell bodies in this region include: two cell bodies of the PVC interneuron (PVCL and PVCR) which are post-synaptic to VD motor neurons in the VNC and are known to regulate locomotion; and the motor/interneuron DVB which located in the dorso-rectal ganglion and sends commissures into the PAG and VNC. No staining was observed in negative control experiments.

**Figure 3:**
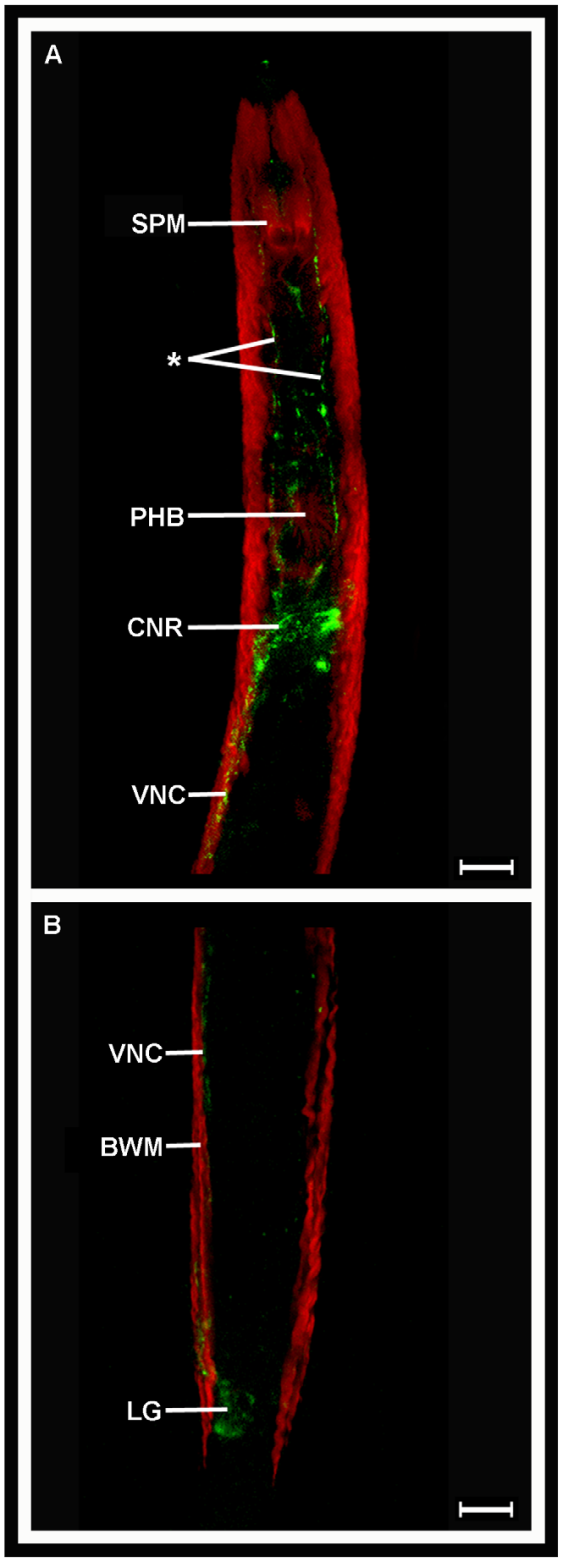
*Globodera pallida* FLP-32 (AMRNALVRFa) is localised in the nervous system of pre-parasitic juveniles. Confocal microscopy images of *G. pallida* FLP-32 (AMRNALVRFa) immunoreactivity (IR; green; A) demonstrate staining in the circumpharyngeal nerve ring (CNR) posterior to the pharyngeal bulb (PHB), and in nerve processes (*) running anteriorly towards the stylet protractor muscles (SPM). IR is also evident in the ventral nerve cord (VNC; A) which emanates from the CNR and runs parallel to the body wall muscle (BWM; red) into the tail where a group of AMRNALVRFa-positive cell bodies are located in close proximity to the lumbar ganglia (LG; B). Scale bars = 75 µm.

A custom raised antiserum directed against the single peptide encoded by *Gp-flp*-32, AMRNALVRFamide, was used to localise *Gp*-FLP-32 using ICC in *G. pallida* J2s. The overall pattern of *Gp*-FLP-32 localisation was similar to the expression pattern of *Gp-flp*-32 exhibited in ISH experiments, comprising extensive AMRNALVRFamide immunostaining within the nervous system of *G. pallida* (see Fig. 3A and B). Strong immunoreactivity was visualised within the CNR, with AMRNALVRFamide-immunopositive nerve processes running both anteriorly and posteriorly from the CNR towards the stylet protractor muscles and the VNC respectively (Fig. 3A). While some staining within the CNR was diffuse as previously noted in ISH experiments, there were distinct accumulations of immunoreactivity on both the ventral and dorsal sides of the nerve ring (see Fig. 3A). In addition there was an accumulation of immunopositive staining, albeit weaker than the anterior staining, in the posterior of the nematode close to the tip of the tail (see Fig. 3B). Again this was concurrent with the positioning of *Gp-flp*-32 expression in the ISH experiments, which would suggest *Gp*-FLP-32 immunoreactivity in cell bodies of the LG.

In this study, *Gp-flp*-32 expression was demonstrated in key neuronal processes involved in nematode motor-control, and when compared to technique-matched ISH data previously published for *Gp*-*flp*-6,-12,-14 and -18 [17], *Gp-flp*-32 expression is much more extensive. As such, it is not unreasonable to suggest that *Gp-flp*-*32* plays a broad role in the neuronal control of *G. pallida* motor function.

### Silencing of *Gp-flp*-32 increases the migration rate of *G. pallida* pre-parasitic J2s

**Figure 4:**
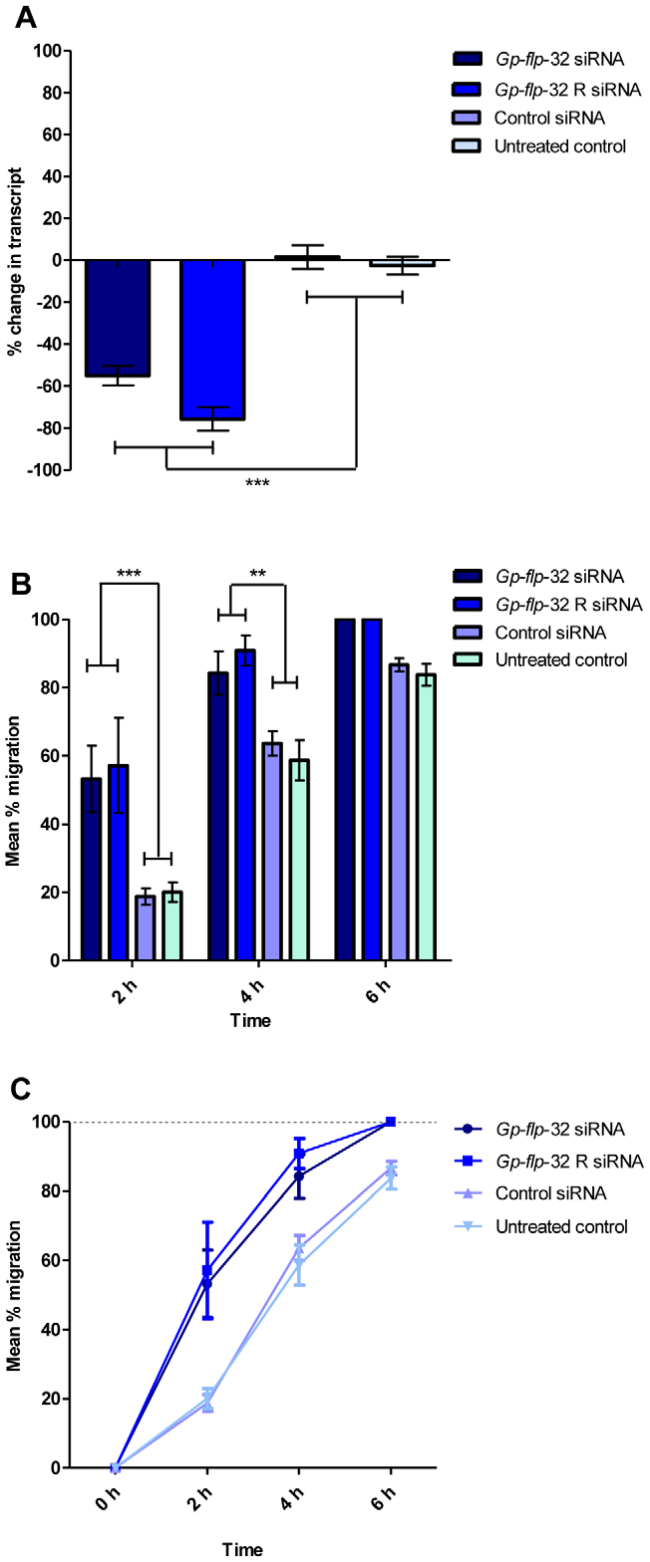
*Globodera pallida flp*-32 (*Gp-flp*-32) and FLP-32 receptor (*Gp-flp*-32R) silenced nematodes exhibit accelerated migration rates. siRNA soaking reduces *Gp-flp*-32 and *Gp-flp*-32R transcript levels by 55% and 75% respectively (A) compared to controls (control siRNA and untreated worms; *P*<0.001). Post-RNAi *Gp-flp*-32 and *Gp-flp*-32R silenced worms displayed an increased speed of migration down a vertical sand column when compared to control worms (B and C). After 4 h migration 84% of *Gp-flp*-32 and 91% of *Gp-flp*-32R worms had migrated compared to 59% and 64% of control siRNA and untreated worms respectively (P<0.01; B and C), after 6 h all *Gp-flp*-32 and *Gp-flp*-32R silenced worms had completed migration (B and C). There was no significant difference in the migratory ability of *Gp-flp*-32 and *Gp-flp*-32R silenced worms over the 6 h migration period (C).

Here we used RNAi soaking experiments, measurements of post-silencing changes in *Gp-flp*-32 transcript levels, and bioassays to assess nematode phenotype, in an attempt to elucidate the role of *flp*-32 in PCN. Consistent and statistically significant reduction in target transcript (quantified as ΔΔC_t_ of *Gp-flp*-32 transcript relative to *Gp-ace* reference transcript) of 55.1±4.6% (n = 3) was achieved in *Gp-flp*-32 siRNA treated worms when compared to untreated worms (*P*<0.001, q = 8.988) and non-native control siRNA treated worms (*P*<0.001, q = 9.716; see Fig. 4A).

Post-RNAi, worm phenotype was assessed by visual observation; worms in all control treatments appeared normal. However, *Gp-flp*-32 siRNA treated worms exhibited an increased frequency of normal sinusoidal movement whereby they appeared to move faster than control worms. This phenotype was quantified through employment of a sand column migration time-course assay [7], where worms were counted every 2 hours (h) as they migrated down a vertical sand column during a 6–8 h period. This demonstrated that *Gp-flp*-32 siRNA treated worms migrated significantly faster than untreated worms (2 h, 53.2±9.7% vs 20.1±2.9% migration respectively, *P*<0.001; 4 h, 84.3±6.3% vs 58.7±5.9% migration respectively, *P*<0.01; n = 3; see Fig. 4B and C) and control siRNA treated worms (2 h, 53.2±9.7% vs 18.8±2.4% migration respectively, *P*<0.001; 4 h, 84.3±6.3% vs 63.6±3.6% migration respectively, *P*<0.01; n = 3; see Fig. 4B and C). At the 6 h migration time point fewer untreated (83.7±3.2%), and non-native control siRNA treated (86.6±2.0%) worms had successfully migrated relative to *Gp-flp*-32 siRNA treated worms; control worms took a further 2 h to complete migration.. During the migration experiment, untreated and control siRNA treated worm migration did not differ significantly at any time (*P*>0.05; n = 6; see Fig. 4C). Together these data suggest that *Gp-flp*-32 expresses an inhibitory neuropeptide, which, when silenced, induces an increase in locomotory activity. The marked stimulation of J2 migration rate following *Gp*-*flp-32* silencing was achieved with only a 55% reduction in transcript, suggesting that the encoded FLP has profound depressive effects on locomotion in wild type worms. These data differ from all published RNAi studies on *flp* gene function in PPNs which are characterized by the induction of phenotypes encompassing unusual body posture, slower movement and/or paralysis [7], [21].

**Figure 5:**
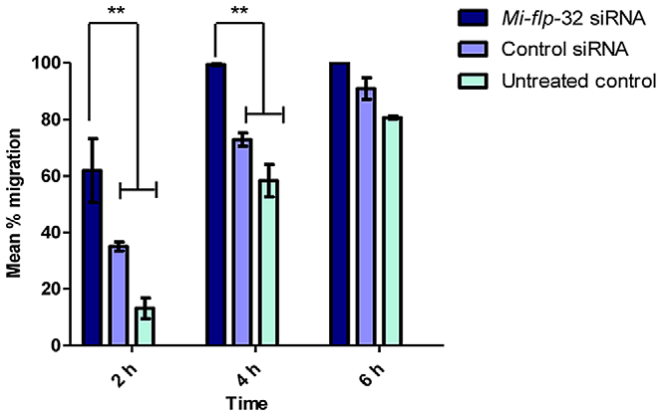
*Meloidogyne incognita flp*-32 (*Mi-flp*-32) silenced nematodes exhibit accelerated migration rates. Post-RNAi *Mi-flp*-32 silenced worms displayed an increased speed of migration down a vertical sand column when compared to control worms. After 2 h migration 62% of *Mi-flp*-32 silenced worms had migrated compared to 35% and 13% of control siRNA and untreated worms, respectively (*P*<0.01; *P*<0.001); after 4 h 99% of *Mi-flp*-32 silenced worms had migrated compared to 73% and 58% of control siRNA and untreated worms respectively (*P*<0.01; *P*<0.001); after 6 h all *Mi-flp*-32 silenced worms had completed migration. There was no significant difference in the migratory ability of control siRNA and untreated worms over the 6 h migration period.

To ascertain if this increased rate of migration in response to *flp*-32 silencing is mirrored by other PPNs, the same migration experiments were performed on the pre-parasitic J2 stage of the root knot nematode *Meloidogyne incognita*. In these experiments worms were pre-treated with an siRNA targeting *M. incognita flp*-32 (*Mi-flp*-32; GenBank accession number CN443314; [11]) with controls as described above. *flp*-32 silenced pre-parasitic *M. incognita* migrate more rapidly than untreated or siRNA control treated worms, mirroring the phenotype of *Gp-flp-32* silencing *G. pallida* J2s (see Fig. 5).

These data show that *flp*-32 plays a key role in the modulation of normal locomotory behaviour in pre-parasitic J2s of two major groups of plant endoparasitic nematodes. These observations are consistent with the hypothesis that FLP-32 depresses locomotory behaviour in wild-type pre-parasitic J2s.

The role of *Gp-flp*-32 is consistent with its expression pattern; *Gp-flp*-32 was identified along the VNC in the cell bodies of DD and VD-like motor neurons, which in *C. elegans* control sinusoidal movement through their innervation of dorsal and ventral muscles, respectively [19], [20]. DD motor neurons relax dorsal muscles during ventral muscle contraction [22], and are believed to regulate the wave amplitude of sinusoidal movement [23]. The DD and VD motor neurons, responsible for the relaxation of dorsal and ventral muscles during sinusoidal movement, could do so in part due to the action of FLP-32.

While the evidence presented here strongly suggests a locomotory role for *Gp-flp*-32, this does not discount the possibility that *Gp-flp*-32 may also regulate other processes in PPNs. Many of the currently available *in vitro* assays for post-RNAi phenotype analysis in PPNs are designed to assess the ability of worms to migrate and move normally, such that disruption to processes such as egg laying or larval development would not be recorded. With this in mind, we employed a potato plant infection assay to determine if *Gp-flp*-32 silenced worms displayed infection-associated phenotypes.

### Silencing of *Gp-flp*-32 enhances the rate of potato root infection by *G. pallida* J2s

To probe the function of *Gp-flp*-32 further, the infectivity of *Gp-flp*-32 silenced J2s was compared in a small-scale potato plant infection assay. A positive control siRNA [directed against transcript encoding acetylcholinesterase (*Gp-ace*); GenBank accession number FJ499505] which displays reduced locomotory activity post-RNAi (unpublished data) was employed, in addition to the standard untreated and non-native siRNA controls described above. The purpose of this positive siRNA control was to demonstrate the effect of reduced locomotory ability on nematode infection rate. RNAi treated nematodes were applied to the sand covering the root network of 2 week old potato plants, and after a period of 4 days, plant roots were analysed for the presence of nematodes.

**Figure 6:**
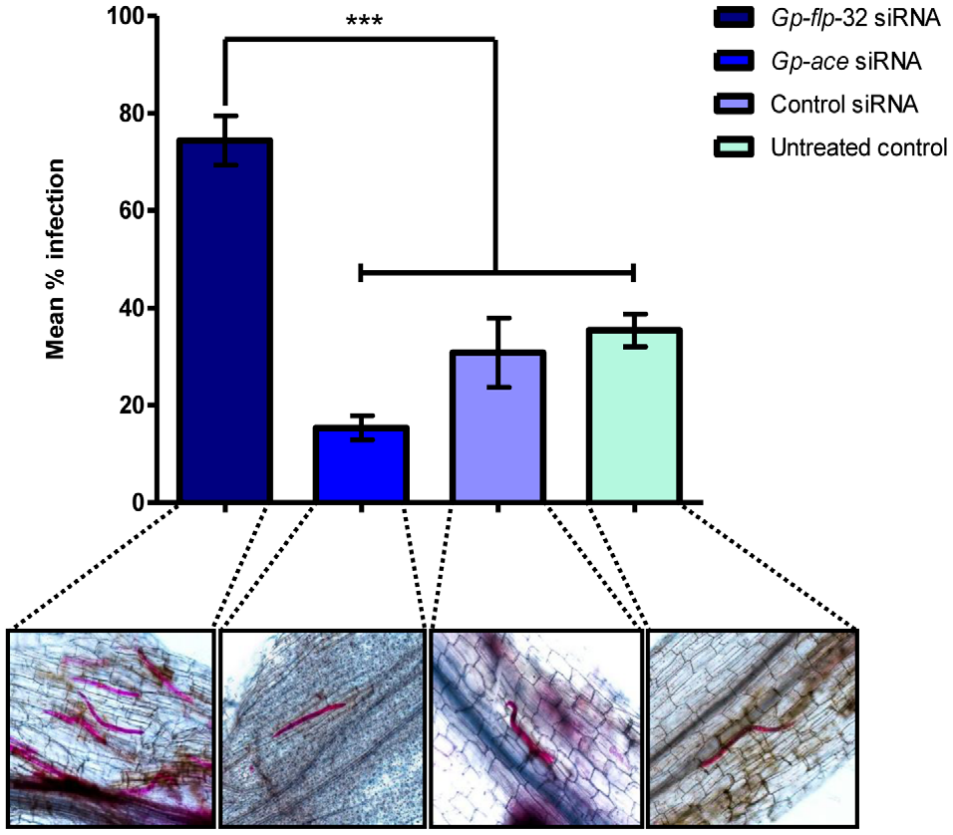
The rate of potato plant root infection is enhanced in *Gp-flp*-32-silenced *Globodera pallida* J2s. Infection rates of *Gp-flp*-32, *Gp-ace* (positive control siRNA), control siRNA (non-native negative control) and untreated worms identified by acid fuschin staining in the roots of potato plants challenged with nematodes post-RNAi. *Gp-flp*-32 siRNA treated worms show a significantly higher mean infection rate of 74% compared to control siRNA (31%) and untreated (35%) worms (*P*<0.001) as shown by the graph and representative light microscopy images of pink acid fuschin stained nematodes in root segments. There was no significant difference in the mean infection rate of *Gp-ace* siRNA, control siRNA and untreated worms.

*Gp-flp*-32 siRNA treated worms displayed a significantly higher mean infection rate of 74.4±5.0% (n = 4) compared to untreated (35.4±3.4%, *P*<0.001, q = 7.72; n = 8), non-native control siRNA (30.8±7.1%, *P*<0.001, q = 8.18; n = 6), and positive control siRNA (15.4±2.5%, *P*<0.001, q = 10.67; n = 5) treated worms (see Fig. 6). When compared, the infection rates of untreated and non-native siRNA treated controls were not significantly different (35.4±3.4% vs 30.8±7.1% infection, respectively; *P*>0.05, q = 1.03; Fig. 6).

This assay confirmed that the increased migration rate displayed by *Gp-flp*-32 silenced worms translated to increased plant root infection rate, i.e. the worms migrate to the root faster and/or infect the root more quickly. Whilst this may reflect an enhancement in sensory ability further improving the chances of host location success, this is unlikely since *Gp-flp*-32 is not localised in areas of the worm associated with chemoreception.

The nature of the *Gp-flp*-32 RNAi phenotype raises a question regarding the ability of worms to sustain their migratory and invasion activities. It is possible that increased rates of migration and infection would more rapidly deplete the finite energy reserves in these non-feeding J2s, resulting in premature death. This possibility was investigated using Oil Red O lipid staining [24], [25] and assessment of lifespan in *Gp-flp*-32 silenced J2s over a 14 day period following RNAi. This assay did not reveal increase in lipid depletion or death rates in *Gp-flp*-32-RNAi treated worms compared to controls (data not shown).

### A novel *Gp-flp*-32 receptor is expressed in *G. pallida*

**Figure 7:**
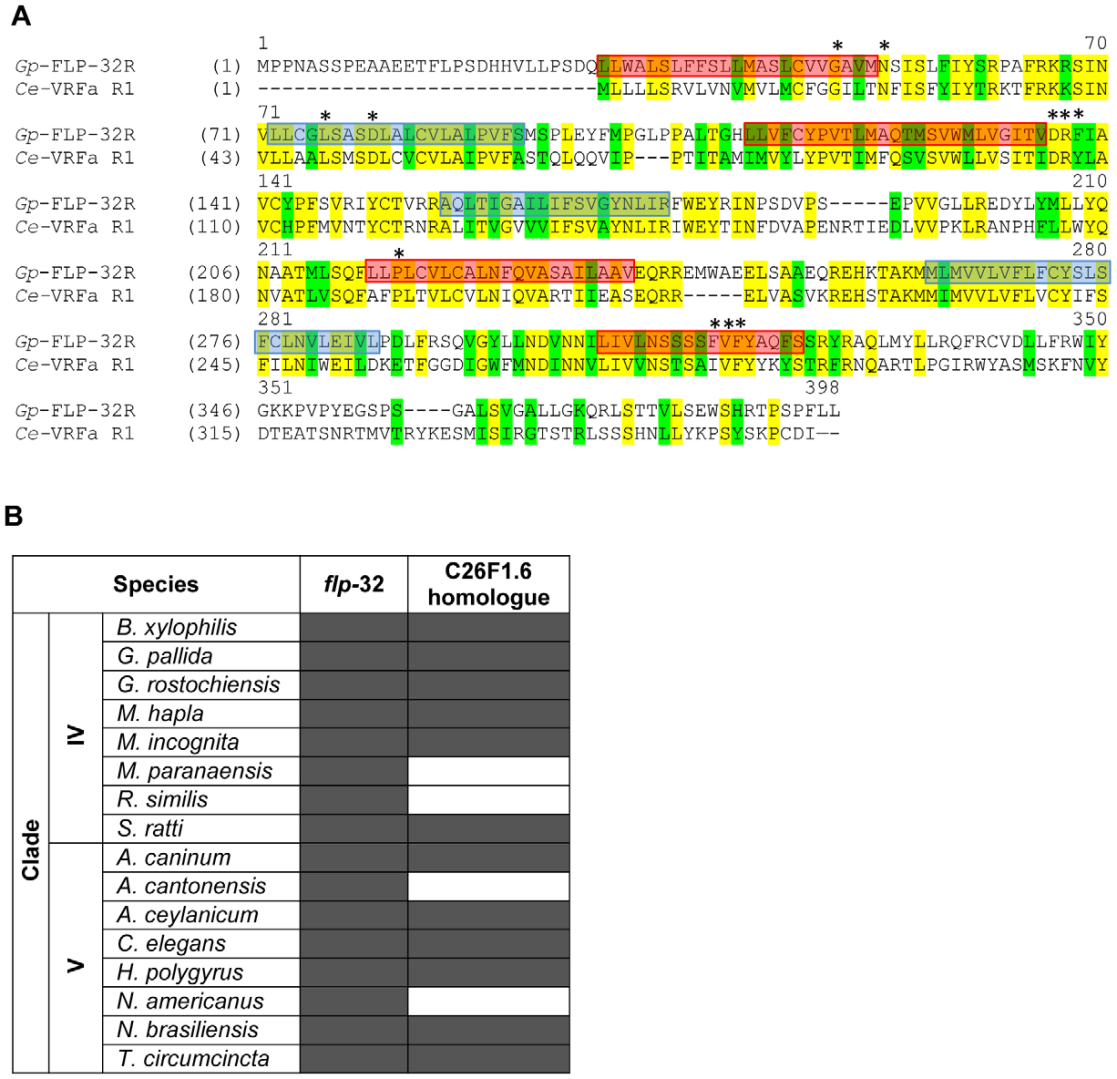
A novel *Gp-flp*-32 receptor (*Gp-flp*-32R) is expressed in *Globodera pallida* J2s and is conserved across the nematode phylum. A novel *Gp-flp*-32R which shows close homology to the *C. elegans* VRFa receptor 1 (C26F1.6) is expressed in *G. pallida* (A). The 389 amino acid protein has seven transmembrane helices; outside to inside helices are boxed in red; inside to outside helices are boxed in blue (A). 11 conserved residues common to rhodopsin family GPCRs are denoted by asterisks (A). Interrogation of available nematode EST, genomic and transcriptomic datasets show that at least 12 of the 16 nematode species from clades IV and V which express *flp*-32 also express a homologue of the *Gp-flp*-32R (B), demonstrating inter-clade conservation of the *flp*-32 receptor. *B. xylophilis: Bursaphelenchus xylophilus; G. pallida: Globodera pallida; G rostochiensis: Globodera rostochiensis; M. hapla*: *Meloidogyne hapla*; *M. incognita*: *Meloidogyne incognita*; *M. paranaensis: Meloidogyne paranaensis; R. similis: Radopholus similis; S. ratti: Strongyloides ratti; A. caninum: Ancylostoma caninum; A. cantonensis: Ancylostoma cantonensis; A. ceylanicum; Ancylostoma ceylanicum; C. elegans: Caenorhabditis elegans; H. polygyrus: Heligmosomoides polygyrus; N. americanus*: *Necator americanus; N. brasiliensis: Nippostrongylus brasiliensis; T. circumcincta: Teladorsagia circumcincta.*

Database mining facilitated the identification of a putative FLP-32 receptor in *G. pallida*, orthologous to the *C. elegans* VRFa receptor R1 (C26F1.6; see Fig. 7A). RACE PCR and sequencing confirmed the sequence of the putative *G. pallida flp*-32 receptor (*Gp-flp*-32R). Primers designed to confirm the open reading frame of *Gp-flp*-32R generated a 1,170 nucleotide cDNA sequence (GenBank accession number JQ685132), encoding a 389 aa protein (Fig. 7A). *Gp-flp*-32R encodes seven transmembrane helices and conserved residues at positions 48, 52, 76, 80, 136–138, 222, 273 and 318–320; the 136–138 sequence (DRF) is a common variation on the DRY motif at the cytosolic end of the third transmembrane helix and is common to rhodopsin-like GPCRs (see Fig. 7A). In a reciprocal tBLASTn search of the *C. elegans* non-redundant nucleotide and protein database, *C. elegans* C26F1.6 was returned as the top scoring hit (53% identity to *Gp-flp*-32R). Further interrogation of the *G. pallida* EST database (GenBank) and genome assembly (Wellcome Trust Sanger Institute November 2010 supercontig assembly) between August and October 2011 with *C. elegans* C26F1.6 did not reveal additional homologous transcripts. BLAST searches of all available nematode EST, genomic and transcriptomic resources identified 12 C26F1.6 GPCR homologues from the 16 species which express FLP-32 encoding transcripts, spanning clades IV and V (Fig. 7B).

Until now orthologues of the deorphanised *C. elegans* FLP GPCRs have not been reported in a parasitic nematode. As a result of the lack of information regarding parasitic nematode neuropeptide receptors, *C. elegans* represents the sole and limited source of GPCR functional data available for nematodes. Nevertheless, combining *C. elegans* receptor-ligand pairing data with sequence data homologies, appropriate expression patterns and matching RNAi phenotypic readouts can facilitate the functional characterisation of peptide-GPCR relationships in parasites.

### Silencing of a putative *Gp-flp*-32R also elicits increased migration rates in non-parasitic J2 stage *G. pallida*

The identification of a putative *Gp-flp*-32R candidate has facilitated the application of functional characterisation tools to (i) confirm the identity of *Gp-flp*-32R as a FLP-32 activated receptor, and (ii) further probe the neuromodulatory role of *Gp-flp*-32 and *Gp-flp*-32R in *G. pallida*. To achieve this we duplicated the RNAi, qPCR, and migration bioassay experiments previously employed for *Gp-flp*-32 characterisation on *Gp-flp*-32R.

Consistent and statistically significant reduction in target transcript (quantified as ΔΔC_t_ of *Gp-flp*-32R transcript relative to *Gp-ace* reference transcript) of 75.7±5.6% (n = 3) was achieved in *Gp-flp*-32R siRNA treated worms when compared to untreated worms (*P*<0.001, q = 9.459) and non-native control siRNA treated worms (*P*<0.001, q = 10.02; see Fig. 4A). Following RNAi experiments all worms appeared normal except that *Gp-flp*-32R silenced worms appeared to show an increased frequency of normal sinusoidal movement compared to controls. This phenotype matched that displayed by *Gp-flp*-32 silenced worms. To further probe the nature of this phenotype, sand column migration assays were employed to monitor worm migration every 2 h over a 6-h period.

Phenotype analysis by migration assay showed that *Gp-flp*-32R siRNA treated worms migrated significantly faster than untreated worms (2 h, 57.1±13.9% vs 20.1±2.9% migration respectively, *P*<0.001; 4 h, 90.9±4.4% vs 58.7±5.9% migration respectively, *P*<0.001; n = 3; see Fig. 4B and C) and control siRNA treated worms (2 h, 57.1±13.9% vs 18.7±2.4% migration respectively, *P*<0.001; 4 h, 90.9±4.4% vs 63.6±3.6% migration respectively, *P*<0.001; n = 3; see Fig. 4B and C) for the first 4 h of migration. At the 6 h migration time point fewer untreated (83.7±3.2%), and non-native control siRNA treated (86.6±2.0%) worms had successfully migrated relative to *Gp-flp*-32R siRNA treated worms; control worms took a further 2 h to complete migration. When compared, the time course migration pattern of *Gp-flp*-32 and *Gp-flp*-32R silenced worms was very similar; there was no statistically significant difference between the migration of *Gp-flp*-32 and *Gp-flp*-32R siRNA treated worms over the 6 h migration period (2 h, 53.2±9.7% vs 57.1±13.9% migration respectively, *P*>0.05; 4 h, 84.3±6.3% vs 90.9±4.4% migration respectively, *P*>0.05; 6 h, 100.0% vs 100.0% migration respectively, *P*>0.05; n = 3; see Fig. 4B and C).

Together these data suggest that (i) *Gp-flp*-32R is involved in the maintenance of normal locomotory activity in *G. pallida*, and (ii) *Gp-flp*-32R is likely to be a FLP-32 activated GPCR, as the post-RNAi phenotypes of *Gp-flp*-32R and *Gp-flp*-32 silenced worms are closely matched. These conclusions are further supported by the localisation of *Gp-flp*-32 in neuronal cell bodies linked to the control of locomotion; unfortunately, repeated attempts to localise *Gp-flp*-32R (ISH) were unsuccessful and so we cannot comment on the co-localisation of peptide and receptor.

Heterologously expressed C26F1.6 is activated by two peptides; FLP-7 (TPMQRSSMVRFamide) and FLP-32 (AMRNALVRFamide; [13]). Both of these peptides display a structurally conserved C-terminus in *C. elegans* and many other nematode species (VRFamide). However, in *G. pallida*, *flp*-7 encodes three peptides with an alternative ARFamide C-terminal motif [11]. Functional characterisation of the *C. elegans* VRFa receptor 1 (C26F1.6) identified SMVRFamide as the most active truncated form of the FLP-7 peptide, suggesting that the terminal five amino acids are the most important for receptor activation [13]. This raises a question as to whether the APLDRSA(M/L/I)ARFamide peptides encoded by *G. pallida flp*-7, which do not fit the VRFamide C-terminal model for C26F1.6 activation, would activate the VRFa receptor 1 homologue (*Gp-flp*-32R) in *G. pallida*. Preliminary data from *G. pallida flp*-7 RNAi experiments suggest that *flp*-7 peptide products do not interact with *Gp*-FLP-32R, as post-RNAi phenotypes for *Gp-flp*-7 and *Gp-flp*-32R silenced worms do not match. Further, *G. pallida flp*-7 localisation patterns are distinct from those reported for *Gp-flp*-32 (unpublished data). Based on these observations it seems reasonable to propose that *Gp*-FLP-32 (AMRNALVRFamide) could be the primary, but not necessarily the sole ligand for *Gp*-FLP-32R.

Curiously, *C. elegans* RNAi screens have revealed that VRFa receptor 1 (C26F1.6) silencing is characterised by hyperactive egg laying activity, caused by the blunted inhibitory activity of VRFa receptor 1 ligands in the neuronal circuitry surrounding the reproductive apparatus [26]. However, this is contradictory to evidence that *flps* which encode the most potently active VRFa receptor 1 ligands (TPMQRSSMVRFamide and AMRNALVRFamide; [13]) are localised throughout the nematode nervous system, and are not limited to neurons associated with the reproductive apparatus [18]. Nevertheless, the findings described here clearly demonstrate that the *Gp-flp*-32R has significant importance in the modulation of motor functions in *G. pallida* that include, but are not necessarily limited to, the control of normal locomotory activity.

### Conclusions

Here we report the characterisation and interrogation of FLP-32/FLP-32R function in the PCN, *G. pallida*. The data indicate that this ligand-receptor pair may interact in PPNs to depress normal locomotory activities, modulating migration and plant root infection behaviours. This is the first functional characterisation and putative deorphanisation of a neuropeptide receptor in a parasitic nematode using reverse genetic tools, and fosters the validation of novel neuropeptidergic control targets. These data support the potential candidature of the FLP-32R as a target for agonistic drugs that would slow and potentially disrupt normal parasite locomotory behaviours.

## Materials and Methods

### Nematode culture and hatching

*Globodera pallida* (Pa2/3) were collected from potato plants of the Cara cultivar and maintained at the Agri-Food and Bioscience Institute (AFBI), Belfast, Northern Ireland. Pre-parasitic J2s were hatched from cysts in fresh potato root diffusate at 15°C in complete darkness. Freshly hatched J2s were washed briefly in DEPC-treated spring water and used immediately in experiments.

The roots of greenhouse maintained, susceptible tomato species infected with *M. incognita* were harvested and washed rigorously in water. Egg masses were removed by brief dissolution in sodium hypochlorite (2.5% v/v) and free eggs washed thoroughly in water. Eggs were isolated by sequential washing through a nested sieve series and placed in tomato root diffusate under complete darkness at room temperature. Freshly hatched pre-parasitic J2 worms were recovered and used immediately in experiments.

### Bioinformatic analysis

Basic Local Alignment Search Tool (BLAST) searches of the *G. pallida* genome were performed using the Wellcome Trust Sanger Institute BLAST server at http://www.sanger.ac.uk/cgi-bin/blast/submitblast/g_pallida. BLAST searches for a putative *G. pallida* VRFamide/FLP-32 receptor (*Gp-flp*-32R) candidate were conducted using the *C. elegans* VRFamide activated receptor 1 (C26F1.6; [13]) as a query in a translated nucleotide BLAST (tBLASTn) search of the *G. pallida* November 2010 supercontig assembly with an expect value of 100. The high scoring return sequences in each hit were combined and translated into a single amino acid sequence in six reading frames (http://web.expasy.org/translate/), the correct reading frame was then subjected to reciprocal tBLASTn and protein BLAST (BLASTp) searches of the *C. elegans* non-redundant nucleotide (nr/nt) database at the National Centre for Biotechnology Information BLAST server (http://blast.ncbi.nlm.nih.gov/), using default settings. The identity of the top scoring reciprocal BLAST hit was regarded as the identity of the original *G. pallida* hit. Finally, putative *Gp-flp*-32R hits were analysed for identity and transmembrane domain structure using the online InterProScan (http://www.ebi.ac.uk/Tools/pfa/iprscan/), TMpred (http://www.ch.embnet.org/software/TMPRED_form.html) and HMMTOP (http://www.enzim.hu/hmmtop/) servers. All BLAST searches were performed between April and October 2011. *G. pallida flp*-32 (GenBank accession number CV578361) and *M. incognita flp*-32 (GenBank accession number CN443314) were previously identified through interrogation of ESTs [10], [11].

### Gene characterisation

Messenger RNA was extracted from approximately 300 *G. pallida* J2s using Dynabeads mRNA Direct kit (Life Technologies) according to the manufacturer's instructions. Separate populations of 5′ and 3′ Rapid Amplification of cDNA Ends (RACE)-ready cDNA were generated using the SMARTer RACE cDNA Amplification kit (Clontech), as described by the manufacturer's instructions.

Gene specific primers (GSP) were designed against a putatively assigned *G. pallida flp*-32 EST (*Gp-flp*-32; GenBank accession number CV578361; [11]) and a putative *G. pallida flp*-32R genome hit (GenBank accession number JQ685132; see Table 1), and were used in 50 µl PCR reactions to confirm the expression presence of the gene transcript of interest as follows: 5 µl 10× PCR buffer (Life Technologies), 3 µl MgCl_2_ (50 mM, Life Technologies), 1 µl dNTP mix (10 mM, Promega), 1 µl of each sense and antisense GSP primer (20 µM), 1 µl cDNA template, 0.3 µl Platinum *Taq* DNA Polymerase (5 U/µl, Life Technologies), ddH_2_O to 50 µl. Thermal cycling conditions were as follows: initial denaturation and ‘hot start’ at 94°C for 2 min, followed by 40 cycles of 94°C 1 min, 55°C 1 min, and 72°C for 1 min, and a final extension step of 72°C for 7 min.

**Table 1:** Primer sequences for PCR, *in situ* hybridisation (ISH), and quantitative (q)PCR analysis.

Gene name (primer used)	Oligonucleotide sequence (S: 5′-3′; A: 3′-5′)	Amplicon size (bp)
*Gp-flp*-32 (GSP) S	TCGTCACGAATTGTCTTTGC	249
*Gp-flp*-32 (GSP) AS	CGCCGACAAAAGCTTCAC	
*Gp-flp*-32 (GSP ORF) S	ATGTCCAAATTATGCCGGTTTT	321
*Gp-flp*-32 (GSP ORF) AS	TTGCTCCGGCCAAAGTAAAAG	
*Gp-flp*-32 (RGSP) S	ATTTGTTGCGCGACATTGG	N/A
*Gp-flp*-32 (RGSP) AS	CCGGCATAATTTGGACATTTCG	
*Gp-flp*-32 (ISH) S	TCGTCACGAATTGTCTTTG	201
*Gp-flp*-32 (ISH) AS	AGCGTACGAGCGCATTTC	
*Gp-flp*-32 (qPCR) S	TCGTCACGAATTGTCTTTGC	108
*Gp-flp*-32 (qPCR) AS	GAAGCACGGCGTAGAAGAAG	
*Gp-flp*-32R (GSP) S	GGGCTCTTTCGCTTTTCTTT	582
*Gp-flp*-32R (GSP) AS	TCAATGCACAAAGCACACAA	
*Gp-flp*-32R (RGSP) S	ACTGCGCGAAGATTACCTGT	N/A
*Gp-flp*-32R (RGSP) AS	TTCATCACAGCTCCGACAAC	
*Gp-flp*-32R (GSP ORF) S	ATGCCCCCAAATGCCTCA	1,170
*Gp-flp*-32R (GSP ORF) AS	TTACAACAAAAAGGGTGACGG	
*Gp-flp*-32R (qPCR) S	ACTGCGCGAAGATTACCTGT	92
*Gp-flp*-32R (qPCR) AS	ATGCACAAAGCACACAAAGC	
*Gp-ace* (qPCR) S	CCAAATTGTGCAGCGTGAAG	96
*Gp-ace* (qPCR) AS	AGAAGTGGTCGTCCTCATCGA	
*Gp-flp-12* (ISH) S	GCTTCTGATGCTGTCGGGTGT	232
*Gp-flp-12* (ISH) AS	GAACTCGAACTTGTTCTTTCGTTT	

(*Gp-flp*-32 GenBank accession number CV578361; *Gp-flp*-32R *G. pallida* genome contig number 1024339; *Gp-ace* GenBank accession number FJ499505; *Gp-flp-12* GenBank accession number CAC32452 [17]; S, sense primer; A, antisense primer).

*Gp-flp*-32 and *Gp-flp*-32R RACE GSPs (RGSP, see Table 2) were used in 3′ RACE and 5′ RACE reactions. The components of 50 µl RACE PCR reactions were as follows: 5 µl 10× PCR buffer (Life Technologies), 3 µl MgCl_2_ (50 mM, Life Technologies), 1 µl dNTP mix (10 mM, Promega), 2.5 µl RACE-ready 5′ or 3′ cDNA template, 5 µl 10× Universal Primer Mix (UPM), 1 µl sense or antisense RGSP (20 µM), 0.3 µl Platinum *Taq* DNA Polymerase (5 U/µl, Life Technologies), ddH_2_O to 50 µl. RACE PCR reactions were carried out using the thermal cycling conditions above with annealing temperatures of 60–65°C. Finally, GSPs were designed to confirm the open reading frame (ORF) of both transcripts (see Table 1.).

**Table 2:** Short interfering (si) RNA sequences.

siRNA name	siRNA sequence (5′-3′)
*Gp-flp*-32 siRNA	AAGCGATGCGAAATGCGCTCG
*Gp-flp*-32R siRNA	TAAGGTCGCAAGTGCAATTTT
*Mi-flp-32* siRNA	AAAAGAAATTCAACGATGGAA
Positive control *Gp-ace* siRNA	AATAGTACGGTAGCCAGTAGG
Non-native negative control siRNA	AACCCTCCCCCTTGACTCATT

*(Gp-flp*-32 GenBank accession number JQ685131; *Mi-flp*-32 GenBank accession number CN443314 *Gp-flp*-32R GenBank accession number JQ685132; *Gp-ace* GenBank accession number FJ499505; Non-native control siRNA (derived from *Macrostomum lignano*) GenBank accession number EG956133).

All PCR reaction products were viewed on a 1% agarose/Tris acetate EDTA (TAE) gel containing 0.0075% (v/v) ethidium bromide (10 mg/ml), and products of the appropriate size were PCR cleaned using Charge Switch PCR Clean-up kit (Life Technologies) or gel purified with Purelink Quick Gel Extraction kit (Life Technologies). Products were cloned into the pCR 2.1 TOPO vector in One Shot Chemically Competent TOP10 *Escherichia coli* (Life Technologies). At least three individual clones (per PCR product) were sequence verified by GATC Biotech (http://www.gatc-biotech.com). Return sequences were analysed using Vector NTI Advance Alignx (Life Technologies).

### siRNA synthesis and soaking

siRNAs were designed against each target transcript (*Gp-flp*-32, GenBank accession number JQ685131; *Gp-flp*-32R, GenBank accession number JQ685132; *Mi-flp-32*, GenBank accession number CN443314), a species specific positive control (*Gp-ace*, GenBank accession number FJ499505), and a non-native negative control derived from the free-living flatworm *Macrostomum lignano* (GenBank accession number EG956133; see Table 2.). siRNAs were synthesised using the *Silencer* siRNA Construction Kit (Ambion, supplied by Life Technologies) according to the manufacturer's instructions, eluted in DEPC treated spring water and stored in 10 µl aliquots at −80°C until use.

Approximately 500 *G. pallida* (or *M. incognita*) J2s were soaked in 0.1 mg/ml target siRNA (*Gp-flp*-32 or *Gp-flp*-32R or *Mi-flp-32*), non-native negative control siRNA, or DEPC treated spring water (untreated negative control). All siRNAs were diluted to a final volume of 50 µl in DEPC treated spring water, soaks were carried out in triplicate, and in RNase-free hydrophobically lined microcentrifuge tubes for 24 h at 15°C in complete darkness. Following the 24 h soaking period worms were washed three times in DEPC treated spring water, transferred to a flat bottom microcentrifuge tube for mRNA extraction, or phenotypically assessed in post-RNAi migration or infection assays.

### Post-RNAi transcript analysis

Following siRNA soaking experiments *G. pallida* mRNA was extracted with Dynabeads mRNA Direct kit (Life Technologies), treated with DNase I (Ambion TURBO DNase, Life Technologies), and used as a template for cDNA synthesis using the Applied Biosystems High Capacity RNA-to-cDNA reverse transcription kit (Life Technologies) according to manufacturer's instructions. To assess transcript knockdown, target and housekeeping reference gene transcripts were amplified from each cDNA in triplicate qPCR's using FastStart SYBR Green Master (Roche). All qPCR primers (Table 1.) were designed using Primer3Plus software (http://www.primer3plus.com/) and optimised for working concentration and annealing temperature prior to use with a RotorGene Q 5-plex HRM qPCR instrument (Qiagen). The efficiency of each PCR reaction was calculated using Real-time PCR Miner (http://www.miner.ewindup.info/Version2; [27]) and used in the relative quantification of target gene transcripts by the augmented comparative C_t_ method (ΔΔC_t_; [28]). Changes in target gene transcript abundance were analysed by one-way ANOVA and Tukey's Honestly Significant Difference (HSD) post-test, using GraphPad PRISM Version 5 package for Windows (GraphPad Software, Inc.). Data with probabilities of less than 5% (*P*<0.05) were deemed statistically significant.

### Post-RNAi phenotype analysis

#### Migration assay

Following 24 h RNAi treatments as described above, approximately 500 *G. pallida* (or *M. incognita*) from each treatment were applied in triplicate to glass columns (5 mm internal diameter) sealed at one end by nylon muslin and containing 0.28 g of moistened USGA sand (grain diameter 0.25–1.0 mm) as previously described [7]. Following migration experiments worms unable to complete migration in all treatment groups were washed out of the sand column and counted. Percentages of target siRNA soaked J2s were standardised to 100% and, subsequently, migration percentages of control siRNA and untreated J2s were expressed relative to siRNA soaked worms. The results of migration assays were analysed by two-way ANOVA and Bonferroni post-tests using GraphPad PRISM Version 5 package for Windows (GraphPad Software, Inc.), data with probabilities of less than 5% (*P*<0.05) were deemed statistically significant.

#### Infection assay

Twenty potato plants of the Kerr's Pink cultivar were grown in USGA (US Golf Association) sand under greenhouse conditions (16 h/8 h light/dark cycle) for approximately two weeks until green shoots were clearly visible. Each plant was grown in an individual pot, supported within a larger pot containing gardening peat to maintain moisture levels in the sand. Prior to inoculation with nematodes plants were watered with 30 ml of spring water. After watering, plants were allowed to stand for 30 min after which approximately 600 J2 *G. pallida*, treated with either *Gp-flp*-32 siRNA, *Gp-ace* positive control siRNA, non-native control siRNA, or DEPC treated spring water (n = 5 plants per treatment), were applied to the sand covering the root network 500 µl of spring water. Plants were maintained under greenhouse conditions for a further four days, after which they were analysed for nematode invasion.

Potato plants were removed from the growth medium, roots were gently rinsed in fresh water, removed from the plant, cut into 2 cm segments and subjected to sodium-hypochlorite-acid-fuchsin staining using methods adapted from [29]. Root segments were placed in 1.5% NaOCl for 2 min with regular agitation, soaked in fresh water for 15 min, added to a vial of acid-fuchsin stain (1 ml acid-fuchsin stain [3.5 g acid-fuchsin, 250 ml acetic acid, 750 ml ddH_2_O], 30 ml ddH_2_O), and placed in a beaker of boiling water for a further 15 min. Roots were de-stained in a vial of acidified glycerin (glycerol acidified with a few drops of 5 N HCl) in boiling water for 50 min. Root sections were mounted on glass microscope slides, and viewed with an Olympus SZ X16 microscope. Nematodes visible within root segments were counted and data were analysed by one-way ANOVA and Tukey's Honestly Significant Difference (HSD) post-test using GraphPad PRISM Version 5 package for Windows (GraphPad Software, Inc.). Data with probabilities of less than 5% (*P*<0.05) were deemed statistically significant.

### *In situ* hybridisation

*In situ* hybridisation (ISH) probe templates (201–232 bp) were generated by PCR (cycling conditions and reaction mixtures described above) using GSPs (see Table 1.) against the target transcripts *Gp-flp*-32 and *Gp-flp*-32R, and a positive control (*Gp-flp*-12; [17]). PCR products were viewed and sequence verified as described above. Digoxigenin (DIG)-labelled single stranded DNA (ssDNA) probes with sense and antisense polarity were generated from cDNA templates by the LATE-PCR method [30], in the following reaction: 5 µl 10× PCR buffer (Life Technologies), 3 µl MgCl_2_ (50 mM, Life Technologies), 2 µl DIG dNTP mix (Roche), 1 µl each of sense and antisense primers (20 µM or 1 µM according to polarity of probe), 2 µl corresponding ISH probe template, 0.3 µl Platinum *Taq* DNA Polymerase (5 U/µl, Life Technologies), and ddH_2_O to 50 µl. ISH was carried out according to methods previously described [17]. Hybridised probes were detected with substrate (5-bromo-4-chloro-3-indolyl phosphate/nitro blue tetrazolium tablet; BCIP/NBT, Sigma-Aldrich) for up to 2 h at room temperature. Specimens were mounted on glass slides and photographed using a Leica DFC300FX camera and Leica FW4000 V 1.2 software with a Leica DMR light microscope.

### Immunocytochemistry (ICC)

A polyclonal antiserum was raised to the single peptide encoded by *flp*-32 (anti-AMRNALVRFamide) in guinea pig (Genosphere Biotechnologies, France), N-terminally coupled to KLH and affinity purified. Approximately 1000 freshly hatched *G. pallida* J2s were immunostained using the indirect immunofluorescence technique [31], using methods previously described [32]. Anti-AMRNALVRFamide primary antiserum was used at 1/100 working dilution, and worms were viewed on a Leica AOBS SP2 confocal scanning laser microscope. Controls included the omission of primary antiserum, replacement of primary antiserum with pre-immune serum from the donor species, and pre-adsorption of the primary antiserum with ≥250 ng of AMRNALVRFamide and an additional FLP peptide (NGAPQPFVRFamide). Pre-adsorption in NGAPQPFVRFamide did not alter staining patterns observed.
